# Poor nutrition status associated with low patient satisfaction six months into treatment for head and neck/esophageal cancer treatment: A prospective multicenter cohort study

**DOI:** 10.1002/ncp.11211

**Published:** 2024-09-22

**Authors:** Adrianne M. Widaman, Andrew G. Day, Maggie A. Kuhn, Rupinder Dhaliwal, Vickie Baracos, Merran Findlay, Judith D. Bauer, Marian de van der Schueren, Alessandro Laviano, Lisa Martin, Leah Gramlich

**Affiliations:** ^1^ Department of Nutrition Food Science and Packaging San Jose State University San Jose California USA; ^2^ Department of Otolaryngology University of California Davis Medical Center Sacramento California USA; ^3^ Department of Public Health Sciences Queens' University Kingston Ontario Canada; ^4^ Canadian Nutrition Society Ottawa Ontario Canada; ^5^ Department of Oncology, Cross Cancer Institute University of Alberta Edmonton Alberta Canada; ^6^ Cancer Services Royal Prince Alfred Hospital Camperdown New South Wales Australia; ^7^ Chris O'Brien Lifehouse Camperdown New South Wales Australia; ^8^ Cancer Care Research Unit, Susan Wakil School of Nursing and Midwifery, Faculty of Medicine and Health University of Sydney Camperdown New South Wales Australia; ^9^ Cancer Clinical Academic Group, South Western Sydney Clinical School, Maridulu Budyari Gumal (SPHERE) University of New South Wales Sydney New South Wales Australia; ^10^ Department of Nutrition Dietetics and Food Monash University Clayton Victoria Australia; ^11^ Department of Nutrition Dietetics and Lifestyle School of Allied Health HAN University of Applied Sciences Nijmegen Gelderland the Netherlands; ^12^ Department of Human Nutrition and Health Wageningen University and Research Wageningen Gelderland the Netherlands; ^13^ Department of Translational and Precision Medicine Sapienza University of Rome Rome Italy; ^14^ Department of Medicine University of Alberta Edmonton Alberta Canada

**Keywords:** adult, enteral nutrition, life cycle, nutrition, nutrition assessment, nutrition support practice, nutrition support teams, oncology, outcomes research/quality, public policy, research and diseases

## Abstract

**Background:**

Patient‐reported outcome measures have been associated with survival in oncology patients. Altered intake and malnutrition are common symptoms for patients treated for head and neck cancer and esophageal cancer (HNC/EC). The purpose of this study was to examine the relationship between patient‐reported satisfaction with medical care and nutrition status.

**Methods:**

This prospective cohort study collected data from 11 international cancer care sites.

**Results:**

One hundred and sixtythree adult patients (*n* = 115 HNC; *n* = 48 EC) completed a patient satisfaction questionnaire (the Canadian Health Care Evaluation Project Lite) and were included. HNC/EC patient global satisfaction with medical care was 88.3/100 ± 15.3 at baseline and remained high at 86.6/100 ± 16.8 by 6 months (100 max satisfaction score). Poor nutrition status, as defined by the Patient‐Generated Subjective Global Assessment Short Form, was associated with lower patient satisfaction with overall medical care, relationship with doctors, illness management, communication, and decision‐making 6 months into treatment (*P* < 0.01). There was no difference in global satisfaction between patients who did and did not report swallowing difficulty (*P* = 0.99) and patients with and without feeding tube placement (*P* = 0.36). Patients who were seen by a dietitian for at least one nutrition assessment had global satisfaction with care that was 16.7 percentage points higher than those with no nutrition assessment (89.3 ± 13.8 vs 72.6 ± 23.6; *P* = 0.005)

**Conclusion:**

In HNC/EC patient‐centered oncology care, decreasing malnutrition risk and providing access to dietitian‐led nutrition assessments should be prioritized and supported to improve patient satisfaction and standard of care. Feeding tube placement did not decrease patient satisfaction with medical care.

## BACKGROUND

High‐quality healthcare services have been shown to be essential in preventing and managing disease.^1^ Gaps in medical care have been associated with increased hospital length of stays, hospital readmissions, and mortality rates.^2^, ^3^, ^4^ A crucial part of quality healthcare involves patient experience of the medical care received.^1^ Patient experience is defined as the full array of patient interactions with the healthcare team and includes the effectiveness of healthcare delivery.^5^ Patient‐reported measures are associated with patient satisfaction and health outcomes.^6^, ^7^, ^8^, ^9^ Higher patient satisfaction has been associated with quality medical care and indicates a positive patient experience. Patient experience, including satisfaction, has been minimally studied in people experiencing head and neck cancer and esophageal cancer (HNC/EC).^10^, ^11^ Patients have reported the need for individually tailored information, consistent messages from all members of the healthcare team, and support groups to cope with the physical/psychosocial challenges associated with the disease and treatment side effects.^12^ Studies have highlighted the need for oncology care to include nutrition and swallow care that is timely and follows a patient‐centered approach throughout treatment.^11^, ^12^, ^13^

Based on the National Committee on Quality Assurance Patient‐Centered Specialty Practice Standards, Patient‐Centered Oncology Care was developed to focus on individualized whole‐person care, improving access to care, and enhancing communication and coordination between providers.^14^ Within the Patient‐Centered Oncology Care conceptual framework, the support self‐care process dimensions involve assessing, developing, and supporting the ability of the patient to care for themselves.^15^ An important piece of self‐care is maintaining activities of daily living such as eating and drinking. For patients with HNC/EC, the ability to chew, swallow, and taste is often impaired because of the disease itself and because of side effects from the treatment modalities (surgery, chemotherapy, and radiation therapy [RT]). For these patients, multimodal treatment can result in a significant change to the lifelong ability to eat and drink. These challenges affect the ability of the patient to meet energy, nutrient, and fluid needs, which may result in dehydration, decreased energy and nutrient intake, and unintentional weight loss.^16^ This impact on the ability to consume meals can have a profound impact on the psychological well‐being of patients.^10^, ^11^, ^17^, ^18^, ^19^ To maintain safety and ensure adequate intake, patients with dysphagia often have to change the way that they consume meals. Food texture and beverage consistency may need to be altered. Meal duration is prolonged owing to the need to modify chewing and swallowing techniques. For many patients with HNC/EC, oral intake is not safe or adequate, and a feeding tube (FT) is required to support nutrient intake. Although qualitative research conducted at single sites has suggested potential overarching themes related to patient experience and nutrition/swallow care,^10^, ^11^ it is unclear how these life‐altering changes impact HNC/EC patient satisfaction with medical care.

The purpose of this study was to evaluate the impact of nutrition status on patient satisfaction with the quality of the medical care in a multisite, international group of patients receiving treatment for HNC/EC. In addition, the relationship between patient satisfaction and dysphagia symptoms, FT placement, and nutrition assessment frequency was examined.

## METHODS

### Study design and participants

The International Audit of Nutrition Care Practices in Patients with Foregut Tumors study collected data from an international cohort of patients with HNC/EC for 6 months of oncology treatment and aimed to evaluate the patient experience in conjunction with assessment of adherence to nutrition practice guidelines.^20^ This international prospective cohort study collected data from 11 international outpatient and inpatient multidisciplinary cancer centers in Canada (*n* = 6), Australia (*n* = 2), Italy (*n* = 1), the Netherlands (*n* = 1), and the United States (*n* = 1). Between 2016 and 2018, all new patients with HNC or EC at selected cancer centers were screened for eligibility. Eligibility requirements included those aged ≥18 years and those who had an Eastern Cooperative Oncology Group performance score of ≥4. Participants receiving palliative care were excluded. Patients who chose to participate provided written consent. The study was approved by each cancer center's institutional review board. Enrolled patients were followed through the trajectory of care for 6 months. Baseline (0) data were collected within 1 month of patients’ first introduction to the cancer center setting. Patients were observed during treatment for 6 months (6) from the initial baseline date.

### Satisfaction with quality of care measure

The Canadian Health Care Evaluation Project (CANHELP) Lite questionnaire was developed to measure patient‐reported experience of medical care received during end of life.^21^, ^22^ Researchers have found the questionnaire to have high validity and reliability in advanced life limited illnesses and palliative populations, including patients with metastatic cancer, and to have good internal consistency when measured against the Global Rating of Satisfaction with Care questionnaire.^21^, ^22^ The questionnaire was reviewed by the authors, all experts in HNC/EC nutrition care, and found to be relevant for the HNC/EC population. The questionnaire used in this study consisted of 21 questions and has been described in detail elsewhere.^21^, ^22^ In short, the main outcome, global satisfaction, was based on the response of the patient to one question, “In general, how satisfied are you with the quality of care you received?”

Secondary outcomes of interest included specific domains of satisfaction.^21^, ^22^ The patient relationship with the doctor domain included three questions related to the doctor's personal interest in the patient, the availability of the doctors, and the level of trust the patient had for the doctors. The illness management domain included nine questions related to satisfaction with the competence of staff, control of physical and emotional symptoms, and whether the treatment was consistent with the wishes of the patient. The communication domain included three questions about doctors listening and patients receiving consistent, straightforward, and honest information. The decision‐making domain included three questions related to patient satisfaction with being part of the decision‐making process. Finally, one question measured the patient's level of feeling at peace. Patients were asked to complete the CANHELP‐Lite at baseline and at 6 months.

### Nutrition status

The scored Patient‐Generated Subjective Global Assessment Short Form (PG‐SGA SF) is a nutrition assessment tool that can be used as a screening/monitoring tool for malnutrition and has been validated in oncology patients.^23^, ^24^ The PG‐SGA SF was used for this study and includes four sections: weight history questions (0–1 points possible), food intake questions (0–5 points possible), patient‐reported symptoms impacting nutrition (0–24 points possible), and activities and functions (0–3).^23^ The points from each section were added for a total PG‐SGA SF score. A higher score indicates a higher risk of malnutrition. PG‐SGA SF results collected at the same time as the CANHELP survey (baseline and 6 months) were evaluated.^25^

### Dysphagia symptoms

Patients' self‐reported symptoms of dysphagia. If “difficulty swallowing” was checked on the symptoms section of the PG‐SGA SF, the patient was considered to have symptoms of dysphagia. If the box was not checked, it was assumed that the patient did not have symptoms of dysphagia. Dysphagia status was collected at baseline and 6 months.

### FT placement

The dietitian providing direct patient care or, if there was no dietitian, a member of the multidisciplinary patient care team, documented on a standardized nutrition practice form whether a FT was placed and the reason for enteral nutrition (EN). Respondents selected if EN was given (yes/no), the FT location, and the reason for EN. Options for the reason for the FT included either the proactive approach; a prophylactically placed tube before symptoms/side effects occurred; or the reactive approach, meaning a tube placed in response to symptoms/side effects. The potential factors resulting in a reactive tube feeding placement included weight loss, poor oral intake, a high dose of planned chemotherapy, a high dose of planned radiation, or other. The clinical judgment of the dietitian determined whether the FT placement was proactive or reactive.

### Frequency of nutrition assessments

Because this study was observational with the intent to describe international current practices, the number of nutrition assessments varied by patient. The total number of nutrition assessments completed over the 6‐month study period was documented and compared with the patient satisfaction score.

### Statistical methods

All CANHELP‐Lite items, including global satisfaction, were scaled from “0” for not at all satisfied to “100” for completely satisfied.^22^ Domains were calculated as the average of nonmissing scaled items belonging to the domain. If more than half of the items were missing, we considered the entire domain as missing.

We reported the overall global CANHELP satisfaction question and the domain as averages with SDs, but all testing used rank‐based nonparametric methods. The CANHELP global satisfaction response and domain scores were compared between two groups by the Wilcoxon rank sum test for two groups or by the Kruskal‐Wallis test when more than two groups were compared. The association between tumor, node, metastasis (TNM)–defined disease stage and global satisfaction (both ordinal) was tested by the Cochran‐Mantel‐Haenszel test of nonzero correlation. Spearman correlation was used to test the correlation between the CANHELP scores and age or the PG‐SGA SF score. Sex, isease stage, and modality were compared between groups by the chi‐square test. However, because of the sparse data, the *P* values for disease stage and modality were estimated by simulating the chi‐square statistic 10,000 times under the null hypothesis of independence. The analysis was conducted using SAS Version 9.4 (SAS Institute Inc, Cary, NC, USA). We considered *P* < 0.05 as indicating statistical significance and did not adjust for the multiplicity of tests.

## RESULTS

Table 1 provides the number of patients who participated in the study and who provided satisfaction scores derived from the CANHELP‐Lite patient survey at baseline or 6 months. Of the 163 patients who had at least one CANHELP‐Lite question completed, the average ± SD age was 62.8 ± 10.2 years and 79% were male. Age, sex, TNM‐defined disease stage, and cancer treatment modality were presented by cancer type in Table 2.

**Table 1:** Number of patients with completed the Canadian Health Care Evaluation Project Questionnaire‐Lite survey questions (total enrolled *n* = 170).

Time	Global question or any domain	Global question	Relationship with doctors domain	Illness management domain	Communication domain	Decision‐making domain	Feeling at peace domain
Number of participants	*n*	*n*	*n*	*n*	*n*	*n*	*n*
Ever	163	162	163	162	163	147	161
At baseline	163	162	163	155	161	130	151
At 6 months	135	131	133	124	130	109	128
Change from baseline to month 6	135	131	133	117	128	92	118

**Table 2:** Patient characteristics by cancer type.

	Head and neck (*n* = 115)	Esophagus (*n* = 48)	All (*n* = 163)	*P* value
Age, y				0.404
Mean ± SD	62.5 ± 10.3	63.6 ± 10.1	62.8 ± 10.2	
Sex, *n* (%)				1
Male	90 (78.3)	38 (79.2)	128 (78.5)	
Female	25 (21.7)	10 (20.8)	35 (21.5)	
Adjudicated TNM stage, *n* (%)				<0.001
1	5 (4.3)	0 (0.0)	5 (3.1)	
1B	0 (0.0)	4 (8.3)	4 (2.5)	
2	8 (7.0)	0 (0.0)	8 (4.9)	
2B	0 (0.0)	13 (27.1)	13 (8.0)	
3	18 (15.7)	0 (0.0)	18 (11.0)	
3A	0 (0.0)	7 (14.6)	7 (4.3)	
3B	0 (0.0)	7 (14.6)	7 (4.3)	
3C	0 (0.0)	1 (2.1)	1 (0.6)	
4	0 (0.0)	3 (6.3)	3 (1.8)	
4A	54 (47.0)	0 (0.0)	54 (33.1)	
4B	13 (11.3)	0 (0.0)	13 (8.0)	
4C	3 (2.6)	0 (0.0)	3 (1.8)	
NA	7 (6.1)	9 (18.8)	16 (9.8)	
NS	7 (6.1)	4 (8.3)	11 (6.7)	
Modality, *n* (%)				<0.001
Unknown	19 (16.5)	0 (0.0)	19 (11.7)	
C	0 (0.0)	2 (4.2)	2 (1.2)	
C + S	0 (0.0)	1 (2.1)	1 (0.6)	
None	3 (2.6)	2 (4.2)	5 (3.1)	
R	15 (13.0)	0 (0.0)	15 (9.2)	
R + C	53 (46.1)	7 (14.6)	60 (36.8)	
R + C + S	8 (7.0)	35 (72.9)	43 (26.4)	
R + S	7 (6.1)	0 (0.0)	7 (4.3)	
S	10 (8.7)	1 (2.1)	11 (6.7)	

*Note*: *P* values for age were from the Wilcoxon rank sum test, and categorical variables were tested by the chi‐square test. However, because of sparse data, the *P* value for TNM stage and modality were based on 10,000 Monte Carlo simulations of the chi‐square test under the null hypothesis.

Abbreviations: C, chemotherapy; EC, esophageal cancer; HNC, head and neck cancer; NA, not available; NS, not significant; R, radiotherapy; S, surgery; TNM, tumor, node, metastasis.

Although patient satisfaction tended to be higher in the EC than in the HNC group, baseline and change from baseline to 6 months were not significantly different between groups (Table 3). Therefore, for the remainder of this analysis, we combined the two cancer types. The mean ± SD global satisfaction with medical care (ranging from 0 for not at all satisfied to 100 for completely satisfied) was 88.3 ± 15.3 at baseline and 86.6 ± 16.8 at 6 months with a change of −2.1 ± 16.7 from baseline to 6 months.

**Table 3:** Patient satisfaction with medical care by cancer type at baseline, 6 months into treatment, and the change within each subject.

Month	HNC, (*n*) mean ± SD	EC, (*n*) mean ± SD	All, (*n*) mean ± SD	*P* value
Global satisfaction				
0	(115) 87.2 ± 16.0	(47) 91.0 ± 13.2	(162) 88.3 ± 15.3	0.188
6	(93) 84.9 ± 17.7	(38) 90.8 ± 13.5	(131) 86.6 ± 16.8	0.086
Change	(93) −3.0 ± 16.0	(38) 0.0 ± 18.4	(131) −2.1 ± 16.7	0.409
Relationship with doctors				
0	(115) 85.7 ± 15.4	(48) 87.5 ± 14.8	(163) 86.2 ± 15.2	0.502
6	(94) 83.4 ± 16.3	(39) 89.0 ± 16.1	(133) 85.1 ± 16.4	0.036
Change	(94) −3.8 ± 15.3	(39) 0.7 ± 21.4	(133) −2.5 ± 17.4	0.131
Illness management				
0	(110) 84.0 ± 14.0	(45) 86.3 ± 12.8	(155) 84.6 ± 13.6	0.369
6	(87) 80.2 ± 17.0	(37) 85.8 ± 15.0	(124) 81.8 ± 16.6	0.087
Change	(83) −4.7 ± 13.9	(34) −1.9 ± 16.9	(117) −3.9 ± 14.9	0.345
Communication				
0	(114) 86.5 ± 16.3	(47) 91.5 ± 14.4	(161) 87.9 ± 15.9	0.032
6	(92) 82.8 ± 18.7	(38) 89.5 ± 16.5	(130) 84.7 ± 18.3	0.044
Change	(91) −4.0 ± 15.8	(37) −1.4 ± 20.8	(128) −3.3 ± 17.3	0.454
Decision‐making				
0	(94) 78.4 ± 20.0	(36) 83.7 ± 18.9	(130) 79.9 ± 19.8	0.187
6	(74) 80.0 ± 19.5	(35) 90.5 ± 13.0	(109) 83.3 ± 18.3	0.006
Change	(64) 0.2 ± 22.0	(28) 5.3 ± 17.1	(92) 1.8 ± 20.7	0.286
Feeling at peace				
0	(106) 73.8 ± 24.2	(45) 78.3 ± 27.0	(151) 75.2 ± 25.1	0.146
6	(89) 71.6 ± 27.2	(39) 83.3 ± 24.6	(128) 75.2 ± 26.9	0.013
Change	(82) −3.0 ± 25.6	(36) 1.4 ± 29.2	(118) −1.7 ± 26.7	0.426

*Note*: *P* value by Wilcoxon rank sum test.

Abbreviations: EC, esophageal cancer; HNC, head and neck cancer.

Age, sex, and treatment modality were not associated with the overall global satisfaction item at baseline or at 6 months or in the change from baseline to 6 months (all *P* > 0.15). The mean ± SD global satisfaction by time period and TNM stage is presented in Table 4. There were no significant differences between TNM stages at baseline or in the change from baseline to month 6. However, at month 6, the mean ± SD satisfaction ranged from 82.1 ± 19.6 in TNM stage 4 patients to 96.9 ± 8.5 in TNM stage 2 patients (*P* = 0.0067).

**Table 4:** Global satisfaction by TNM‐defined disease stage and time period.

Period	TNM stage	*n*	Mean	SD
Baseline (*P* = 0.21)	1	9	88.9	13.2
	2	21	91.7	16.5
	3	33	89.4	14.0
	4	73	86.3	16.7
Month 6 (*P* = 0.0067)	1	9	88.9	13.2
	2	16	96.9	8.5
	3	27	89.8	12.5
	4	60	82.1	19.6
Change (*P* = 0.26)	1	9	0.0	0.0
	2	16	1.6	17.0
	3	27	−0.9	16.2
	4	60	−3.8	18.9

*Note*: *P* value by Cochran‐Mantel‐Haenszel test of nonzero correlation.

Abbreviation: TNM, tumor, node, metastasis.

A total of 89 (55%) of the 163 patients received an FT placement at some point during the study period. Patient characteristics and treatment modalities described by the EN group and no EN support group can be found in Table 5. A total of 63% (103/163) of patients received either chemotherapy + RT or chemotherapy + RT + surgery. The EN FT group had a majority of stage 3 and 4 patients (83.1%). A total of 77% of the EN FT group received chemotherapy + radiation or chemotherapy + radiation + surgery as compared with 45.9% of the no EN FT group. Table 6 compares patient satisfaction between patients who received EN and those who did not. Global satisfaction with the quality of medical care, relationship with doctors, illness management, communication, and decision‐making did not differ significantly between groups with the exception of the relationship with doctor domain at baseline in the no EN group (*P* = 0.031). Among the 89 patients with EN FT placements, the majority (*n* = 58; 65%) were considered as reactive placements (Table 7). The global patient satisfaction score in the reactive tube placement group was lower at 6 months, averaging 84.1/100 ± 19.5 as compared with the 92.6/100 ± 13.5 in the proactive placement group (*P* = 0.046). The reasons (multiple reasons accepted) for reactive tube placement included weight loss (71%), poor oral intake (69%), high‐dose chemotherapy (19%), high‐dose RT (26%), extent of tumor (34%), and other (60%). No further significant differences in the CANHELP‐Lite domains between the groups receiving either a reactive or a proactive FT were found (Table 7). The sample was too small to determine any statistically significant differences based on the reason for the tube placement.

**Table 5:** Patient characteristics by EN feeding tube placement status.

	Had EN tube (*n* = 89)	Never had EN tube (*n* = 74)	All (*n* = 163)	*P* value
Age				0.059
Mean ± SD	61.4 ± 10.6	64.6 ± 9.5	62.8 ± 10.2	
Sex, *n* (%)				0.849
Male	69 (77.5)	59 (79.7)	128 (78.5)	
Female	20 (22.5)	15 (20.3)	35 (21.5)	
Adjudicated TNM stage, *n* (%)				0.61
1	2 (2.2)	3 (4.1)	5 (3.1)	
1B	3 (3.4)	1 (1.4)	4 (2.5)	
2	3 (3.4)	5 (6.8)	8 (4.9)	
2B	7 (7.9)	6 (8.1)	13 (8.0)	
3	10 (11.2)	8 (10.8)	18 (11.0)	
3A	6 (6.7)	1 (1.4)	7 (4.3)	
3B	5 (5.6)	2 (2.7)	7 (4.3)	
3C	1 (1.1)	0 (0.0)	1 (0.6)	
4	0 (0.0)	3 (4.1)	3 (1.8)	
4A	30 (33.7)	24 (32.4)	54 (33.1)	
4B	7 (7.9)	6 (8.1)	13 (8.0)	
4C	1 (1.1)	2 (2.7)	3 (1.8)	
NA	7 (7.9)	9 (12.2)	16 (9.8)	
NS	7 (7.9)	4 (5.4)	11 (6.7)	
Modality, *n* (%)				<0.001
Unknown	3 (3.4)	16 (21.6)	19 (11.7)	
C	0 (0.0)	2 (2.7)	2 (1.2)	
C + S	1 (1.1)	0 (0.0)	1 (0.6)	
None	0 (0.0)	5 (6.8)	5 (3.1)	
R	4 (4.5)	11 (14.9)	15 (9.2)	
R + C	38 (42.7)	22 (29.7)	60 (36.8)	
R + C + S	31 (34.8)	12 (16.2)	43 (26.4)	
R + S	6 (6.7)	1 (1.4)	7 (4.3)	
S	6 (6.7)	5 (6.8)	11 (6.7)	

*Note*: *P* values for age were from the Wilcoxon rank sum test, and categorical variables were tested by the chi‐square test. However, because of sparse data the *P* value for TNM stage and modality were based on 10,000 Monte Carlo simulations of the chi‐square test under the null hypothesis.

Abbreviations: C, chemotherapy; EN, enteral nutrition; NA, not available; NS, not significant; R, radiotherapy; S, surgery; TNM, tumor, node, metastasis.

**Table 6:** Relationship between enteral nutrition support and head and neck cancer and esophageal cancer patient satisfaction with quality of medical care.

Month	Received enteral nutrition, (*n*) mean ± SD	No enteral nutrition, (*n*) mean ± SD	All, (*n*) mean ± SD	*P* value
Global satisfaction (0–100)				
0	(89) 88.5 ± 15.1	(73) 88.0 ± 15.7	(162) 88.3 ± 15.3	0.864
6	(71) 87.3 ± 17.9	(60) 85.8 ± 15.5	(131) 86.6 ± 16.8	0.364
Change	(71) −1.4 ± 17.4	(60) −2.9 ± 16.0	(131) −2.1 ± 16.7	0.627
Domains of satisfaction				
Relationship with doctors				
0	(89) 88.5 ± 14.6	(74) 83.5 ± 15.6	(163) 86.2 ± 15.2	0.031
6	(72) 85.1 ± 17.7	(61) 85.0 ± 14.8	(133) 85.1 ± 16.4	0.597
Change	(72) −4.7 ± 18.0	(61) 0.1 ± 16.4	(133) −2.5 ± 17.4	0.106
Illness management				
0	(84) 85.2 ± 12.9	(71) 83.9 ± 14.5	(155) 84.6 ± 13.6	0.731
6	(66) 82.1 ± 16.6	(58) 81.5 ± 16.7	(124) 81.8 ± 16.6	0.906
Change	(61) −4.5 ± 15.4	(56) −3.2 ± 14.4	(117) −3.9 ± 14.9	0.704
Communication				
0	(87) 89.7 ± 14.4	(74) 85.9 ± 17.3	(161) 87.9 ± 15.9	0.222
6	(70) 84.9 ± 19.1	(60) 84.6 ± 17.5	(130) 84.7 ± 18.3	0.773
Change	(68) −4.7 ± 17.9	(60) −1.7 ± 16.7	(128) −3.3 ± 17.3	0.535
Decision‐making				
0	(69) 79.8 ± 19.9	(61) 80.0 ± 19.8	(130) 79.9 ± 19.8	0.977
6	(58) 83.4 ± 18.8	(51) 83.2 ± 17.8	(109) 83.3 ± 18.3	0.944
Change	(48) 0.1 ± 21.5	(44) 3.6 ± 19.9	(92) 1.8 ± 20.7	0.157
Feeling at peace				
0	(79) 73.7 ± 27.4	(72) 76.7 ± 22.3	(151) 75.2 ± 25.1	0.754
6	(71) 75.7 ± 27.0	(57) 74.6 ± 26.9	(128) 75.2 ± 26.9	0.772
Change	(62) −1.2 ± 26.6	(56) −2.2 ± 27.1	(118) −1.7 ± 26.7	0.730

*Note*: *P* value by Wilcoxon rank sum test. Global patient satisfaction and domains were measured using the Canadian Health Care Evaluation Project Questionnaire‐Lite score (0 min–100 max). 0 and 6 = group mean at each time point; change = baseline–6 month score within each subject.

**Table 7:** Satisfaction by reactive enteral tube placement vs proactive enteral tube placement.

Month	Reactive tube placement, (*n*) mean ± SD	Proactive tube placement, (*n*) mean ± SD	All patients, (*n*) mean ± SD	*P* value
Global satisfaction (0–100)				
0	(58) 87.9 ± 16.4	(31) 89.5 ± 12.5	(89) 88.5 ± 15.1	0.909
6	(44) 84.1 ± 19.5	(27) 92.6 ± 13.5	(71) 87.3 ± 17.9	0.046
Change	(44) −2.8 ± 17.2	(27) 0.9 ± 17.7	(71) −1.4 ± 17.4	0.321
Domains of satisfaction				
Relationship with doctors				
0	(58) 86.1 ± 15.8	(31) 93.0 ± 10.8	(89) 88.5 ± 14.6	0.047
6	(45) 85.3 ± 16.8	(27) 84.7 ± 19.4	(72) 85.1 ± 17.7	0.833
Change	(45) −1.7 ± 18.3	(27) −9.7 ± 16.5	(72) −4.7 ± 18.0	0.107
Illness management				
0	(53) 83.5 ± 14.1	(31) 88.3 ± 10.2	(84) 85.2 ± 12.9	0.155
6	(41) 81.2 ± 15.2	(25) 83.6 ± 18.9	(66) 82.1 ± 16.6	0.282
Change	(36) −3.3 ± 14.3	(25) −6.2 ± 17.0	(61) −4.5 ± 15.4	0.924
Communication				
0	(57) 88.3 ± 15.7	(30) 92.2 ± 11.4	(87) 89.7 ± 14.4	0.597
6	(43) 85.5 ± 17.6	(27) 84.0 ± 21.4	(70) 84.9 ± 19.1	0.873
Change	(42) −3.0 ± 19.0	(26) −7.4 ± 15.9	(68) −4.7 ± 17.9	0.475
Decision‐making				
0	(43) 78.1 ± 21.7	(26) 82.5 ± 16.8	(69) 79.8 ± 19.9	0.562
6	(34) 84.9 ± 17.7	(24) 81.4 ± 20.5	(58) 83.4 ± 18.8	0.543
Change	(26) 1.9 ± 22.8	(22) −2.1 ± 20.1	(48) 0.1 ± 21.5	0.521
Feeling at peace				
0	(53) 70.8 ± 28.9	(26) 79.8 ± 23.5	(79) 73.7 ± 27.4	0.193
6	(44) 72.7 ± 27.4	(27) 80.6 ± 26.3	(71) 75.7 ± 27.0	0.185
Change	(39) −1.3 ± 29.8	(23) −1.1 ± 20.6	(62) −1.2 ± 26.6	0.844

*Note*: *P* value by Wilcoxon rank sum test. Overall patient satisfaction and subsections were measured using the Canadian Health Care Evaluation Project Questionnaire‐Lite score (0 min–100 max). 0 and 6 = group mean at each time point; change = baseline–6 month score within each subject.

**Figure 1:**
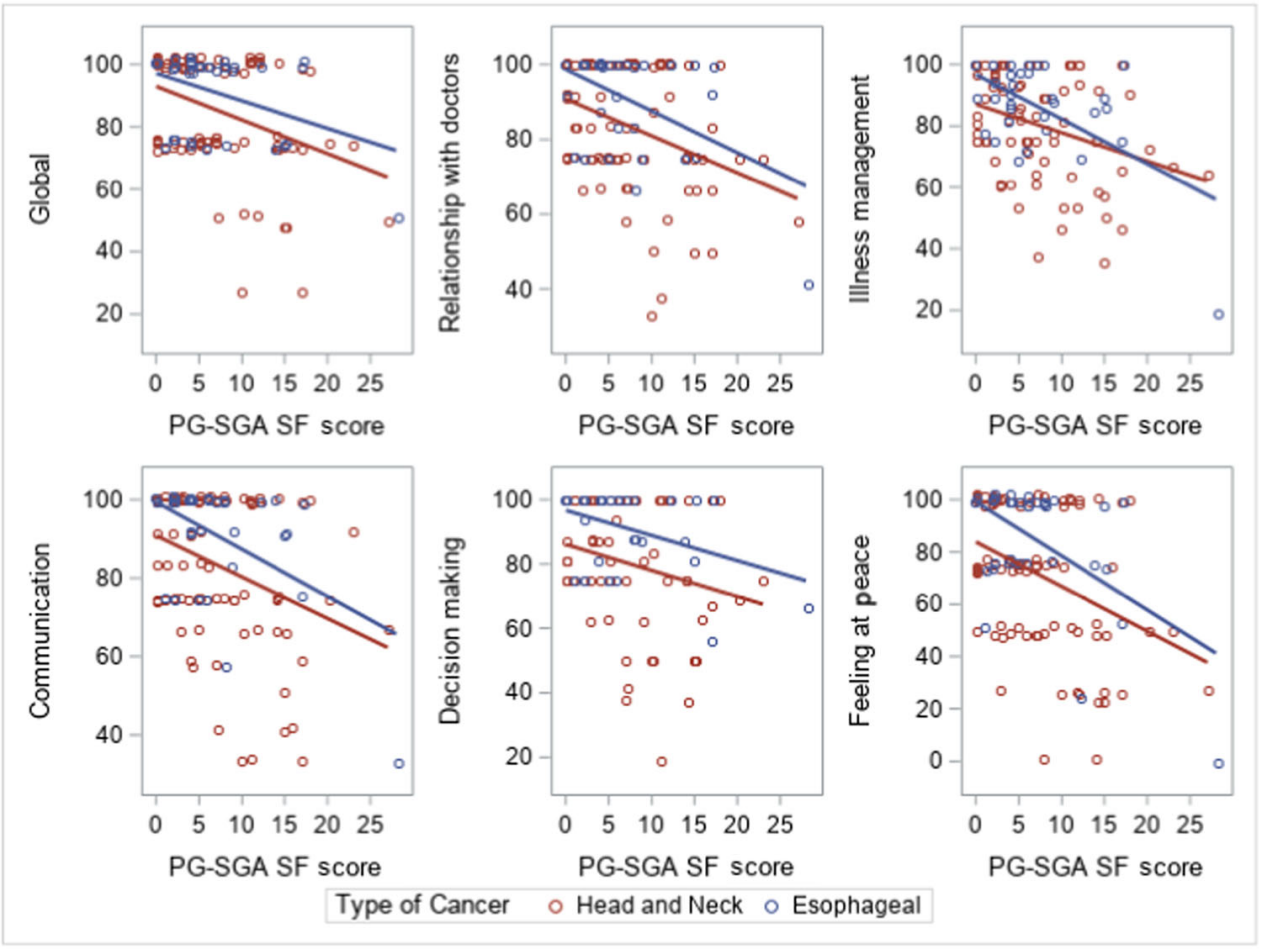
Relationship between PG‐SGA SF score and satisfaction with quality of medical care in foregut tumor patients 6 months into oncology medical care. Correlation at 6 months between the PG‐SGA SF score and Canadian Health Care Evaluation Project Questionnaire were all significant at *P* < 0.01, except decision‐making was *P* = 0.049. In particular, the Spearman rho correlations were as follows: overall satisfaction *r*_s_ = −0.25, relationship with doctors *r*_s_ = −0.29, illness management *r*_s_ = −0.28, communication *r*_s_ = −0.29, decision‐making *r*_s_ = −0.20, and feeling at peace *r*_s_ = −0.28. For the PG‐SGA SF, a higher score indicates a greater risk of malnutrition. PG‐SGA SF, Patient‐Generated Subjective Global Assessment Short Form.

At 6 months, the PG‐SGA SF score was negatively associated (Figure 1) with all the CANHELP‐Lite domains and selected items (all *P* < 0.01 except decision‐making, which was *P* = 0.049). The results mean that patients with greater markers of malnutrition had lower patient satisfaction scores. The Spearman correlation coefficient between the PG‐SGA SF and the global CANHELP score was *r*_s_ = −0.25 (*P* = 0.006). The linear regression lines indicated that the negative association between satisfaction and PG‐SGA SF was fairly consistent in patients with HNC and EC. No difference in satisfaction was found between patients with dysphagia symptoms and those without symptoms (Table 8).

**Table 8:** Six‐month satisfaction by symptoms of dysphagia.

Score/domain (0–100)	Had difficulty swallowing (*n* = 43), (*n*) mean ± SD	Did not have difficulty swallowing (*n* = 81), (*n*) mean ± SD	All patients (*n* = 124),^a^ (*n*) mean ± SD	*P* value
Global satisfaction	(42) 85.7 ± 20.0	(76) 87.2 ± 15.5	(118) 86.7 ± 17.2	0.992
Relationship with doctors	(39) 80.8 ± 22.6	(76) 83.6 ± 18.1	(115) 82.6 ± 19.6	0.725
Illness management	(40) 78.9 ± 19.6	(72) 83.5 ± 15.0	(112) 81.9 ± 16.9	0.313
Communication	(41) 80.5 ± 23.4	(76) 87.8 ± 14.4	(117) 85.3 ± 18.3	0.251
Decision‐making	(37) 80.9 ± 21.4	(60) 85.0 ± 16.6	(97) 83.5 ± 18.6	0.500
Feeling at peace	(41) 69.5 ± 31.4	(76) 78.3 ± 24.3	(117) 75.2 ± 27.2	0.202

*Note*: *P* value by Wilcoxon rank sum test.

^a^ Patients with the difficulty swallowing question answered on the Patient‐Generated Subjective Global Assessment Short Form at 6 months.

The relationship between 6‐month patient satisfaction with care and the number of times a patient was seen for a nutrition assessment was evaluated. Among patients with a 6‐month global satisfaction score and completing at least one nutrition practices data collection form (*n* = 128), the mean global 6‐month satisfaction for the 21 participants with 0 nutrition assessments was 72.6 ± 23.6 vs 89.3 ± 13.8 in the 107 people with one or more assessments. Thus, the mean global satisfaction was 16.6 percentage points (95% CI, 5.6–27.6; *P* = 0.001) greater in patients with at least one nutrition assessment than in patients without any nutrition assessments. Excluding participants with no nutrition assessments (*n* = 107) there was no correlation between satisfaction and number of nutrition assessments.

## DISCUSSION

This study of 163 adult patients with HNC/EC from 11 international sites in five countries demonstrated a high patient satisfaction with medical care received 6 months into oncology treatment (mean global satisfaction 87/100) with only a 2% change from baseline to 6 months. In our study, global satisfaction was 14.8 percentage points higher in patients with stage 2 vs stage 4 disease. Multidisciplinary rounds/conferences have been shown to increase adherence to national guidelines and decrease the time between diagnosis and the start of treatment, thus increasing the quality of care.^26^, ^27^ It is unclear in this study if multidisciplinary medical care contributed to the high patient satisfaction. This sample includes inpatients and outpatients with all four tumor stages increasing the generalizability of the findings to international patients with HNC/EC.

Recently, the relevance and importance of measuring patient‐reported outcomes (PROs) in oncology care has been reviewed.^6^, ^7^, ^8^, ^28^ PRO measurement allows for the evaluation of medical care from a patient perspective, resulting in an opportunity to improve patient‐centered care, outcomes, and possibly survival.^7^, ^8^, ^29^ A prospective cohort study of patients with gastrointestinal cancers found that early changes in PRO measurements were associated with treatment response and survival.^29^ In this study, the CANHELP‐Lite questionnaire measured the patient‐reported experience with medical care. Improving patient satisfaction could be considered a measure for addressing patient‐centered needs. It could also be speculated that increasing patient satisfaction through PROs provides an opportunity to positively impact outcomes of high importance to patients with HNC/EC. Patient‐reported measures ensure patient‐centered medical care and have been positively associated with patient satisfaction, safety, and health outcomes.^6^

This is the first international, prospective study to compare HNC/EC patient satisfaction during the trajectory of standard oncology care (6 months). This study captured a large number of patients with FTs (>50%). A significant main outcome finding was that patients with HNC/EC with FT placement were as satisfied with global medical care as patients who did not receive FTs. A systematic review in all disease types found that enteral tube feeding improved quality of life.^30^ Qualitative studies have highlighted enteral feeding challenges and management requirements with enteral FTs for patients with HNC and their caregivers.^31^ Yet, the findings from this study suggest that these challenges may not negatively impact patient satisfaction with overall medical care, relationships with doctors, illness management, communication with the medical team, or decision‐making.

Because of the location and treatment of HNC/EC, adequate food and beverage intake becomes difficult, and EN FT support may become necessary. The decision to place an FT during HNC/EC treatment and whether to place it prophylactically or in reaction to side effects is complex. A 2013 Cochrane review found insufficient evidence to determine an optimal enteral feeding method.^32^ The 2017 European Society for Clinical Nutrition and Metabolism suggested that prophylactic FT helped maintain nutrition status and avoid treatment interruptions but was limited by insufficient randomized control trial evidence.^33^ The 2021 European Society for Clinical Nutrition and Metabolism guidelines recommended prophylactic FT placement to maintain nutrition status and avoid treatment interruptions when severe radiation‐induced mucositis is expected (ie, a combined radiation and chemotherapy).^34^ Internationally, clinician considerations for FT placement have been reported to include planned treatment, tumor characteristics, nutrition and swallow status, tube dependence risk, psychosocial status, and patient preference.^35^ A secondary finding of this study was that 6 months into treatment, within the FT placed group, patients with prophylactic FT placement were more globally satisfied than patients who received an FT in reaction to worsening sign/symptoms. However, when comparing the patient satisfaction change scores within subjects and satisfaction domains there was no difference. Further study is needed to understand this complex issue. Reactive FT placement may be a marker of higher symptom burden, less supportive care, or less symptom management. It is also possible that the reactive FT group was less prepared for side effects and the possibility of needing enteral feeding.

Literature has suggested that gastrointestinal oncology patient satisfaction with quality of care is independent of morbidity, treatment, and quality of life.^36^ Yet, the results of this study suggested that low patient satisfaction was associated with poor nutrition status. Patients with poor nutrition status 6 months into treatment were less satisfied with the management of their illness, with the relationship with their doctor, with their communication with the healthcare team, and with their ability to be part of decision‐making. Results from this study suggested that nutrition status and patient satisfaction with care were related.^37^ From the patient perspective, nutrition care is not separate from medical care and must be viewed as a vital part of treatment.^38^ Access to quality nutrition care includes screening, assessment, and reassessments that include goals, nutrition prescriptions, implementation of interventions, and evaluation of the effectiveness of the intervention.^20^, ^39^ Registered dietitians provide this level of nutrition care. Yet, it can be argued that in some countries, oncology patients do not have adequate access to nutrition care. A 2019 US national survey of outpatient oncology centers found the average dietitian‐to‐patient ratio to be 1:2308 compared with the recommended 1:120.^39^ International challenges to adequate oncology dietitian staffing needs further study. The use of PROs during treatment has been shown to improve patient‐clinician communication by increasing symptom awareness/discussion and improving multidisciplinary communication.^9^ Further study is needed to determine whether improving nutrition status improves patient satisfaction.

Strong evidence underlies the recommended nutrition screening and nutrition assessment as routine practice for patients with HNC/EC.^40^ However, studies suggest that actual practice does not always match practice guidelines.^20^, ^39^, ^41^, ^42^ One aim of this study was to determine whether patients with HNC/EC who received comprehensive nutrition assessments were more satisfied with global medical care then patients who did not. The observational design of this study allowed clinicians to continue standard practice in their country resulting in 13% of patients not receiving a comprehensive nutrition assessment. These patients were significantly less satisfied with medical care than those who did receive one or more nutrition assessments. A systematic review of randomized control trials found that patients with HNC receiving weekly individualized nutrition counseling during treatment showed consistent improvements in nutrition status, quality of life, treatment interruptions, unplanned hospitalizations, and mortality when compared with controls.^43^ This study did not detect a difference in satisfaction as the frequency of nutrition assessments increased. Further study, using a more sensitive measure, is needed to determine the impact of the frequency of nutrition assessments on patient satisfaction.

This is the first prospective study to evaluate the relationship between patient satisfaction and nutrition/swallowing status/FT placement in an international multisite population of patients with HNC and EC undergoing treatment. Data collection represented actual practice, increasing the generalizability of the findings. This study also has some limitations. Although validated in critically ill populations, the CANHELP‐Lite questionnaire has not been validated in an HNC/EC population undergoing oncology treatment. The large number of statistical tests could have yielded some type I errors, particularly among the differences that were of borderline statistical significance. Conversely, the power of some comparisons may be low because of the limited sample size of some groups. In addition, the definition of proactive and reactive tube placement was based on clinical judgment instead of a strict definition. Finally, it is possible that a more robust measure of dysphagia status may better explain the relationship between patient satisfaction with medical care and dysphagia symptoms. An expert consensus statement on the management of dysphagia in HNC recommended screening for dysphagia before the initiation of cancer therapy using validated screening tools.^44^ In addition, the evaluation of the presence of dysphagia using either a flexible endoscopic evaluation or a video fluoroscopic swallow study was recommended as best practice.^44^

## IMPLICATIONS

Nutrition status is a key to patient experience and satisfaction. From the perspective of patients with HNC/EC, nutrition care is not separate from medical care. Thus, these results justify financially prioritizing dietitian staffing as a potential strategy to increase patient satisfaction with overall medical care.

This study found that poor nutrition status was correlated with lower HNC/EC patient satisfaction with global medical care, relationship with doctors, illness management, communication, and medical decision‐making. Global medical satisfaction was higher among patients who had at least one comprehensive nutrition assessment than those with no nutrition assessments. Policy should reflect comprehensive nutrition assessment for all patients with HNC/EC.^39^

No justification for avoiding enteral tube placement to improve patient satisfaction was found. FT placement did not decrease patient satisfaction with overall medical care scores. Six months into treatment, the FT group was as satisfied as the non‐FT group.

This study adds to the evidence that patient perception of the quality of medical care is impacted by nutrition and feeding. Yet, it can be argued that oncology patients do not have adequate access to nutrition care with a mean registered dietitian–to‐patient ratio of 1:2308 in the US.^39^ With the goal of improving patient satisfaction, hospital systems and oncology leadership should allocate funding for HNC/EC‐specific oncology dietitians as the standard of care.

## AUTHOR CONTRIBUTIONS

Leah Gramlich, Adrianne M. Widaman, Lisa Martin, and Rupinder Dhaliwal conceived and designed the study. Leah Gramlich, Adrianne M. Widaman, Rupinder Dhaliwal, Merran Findlay, Judith D. Bauer, Marian de van der Schueren, Maggie A. Kuhn, and Alessandro Laviano acquired the data. Leah Gramlich, Rupinder Dhaliwal, Vickie Baracos, Merran Findlay, Judith D. Bauer, Andrew G. Day, Marian de van der Schueren, and Adrianne M. Widaman contributed to the data analysis and interpretation. Adrianne M. Widaman, Leah Gramlich, Andrew G. Day, and Maggie A. Kuhn contributed to the writing and editing of the manuscript.

## CONFLICT OF INTEREST STATEMENT

Leah Gramlich received an investigator‐initiated grant from Fresenius Kabi Deutschland and an educational grant from Baxter during the conduct of the study. Rupinder Dhaliwal reports travel support for investigator meeting from Nestlé Health Science Canada and personal fees from Alberta Health Services during the conduct of the study. Alessandro Laviano reports personal fees from Abbott, personal fees from Baxter, personal fees from BBraun, personal fees from Fresenius‐Kabi, personal fees from Nestlé Health Science, and personal fees from Nutricia outside the submitted work. Judith D. Bauer reports personal fees from Nutricia outside the submitted work. Andrew G. Day received funding from Alberta Heath Services for statistical consulting. Vickie Baracos is a consultant to Nestlé Health Sciences and Pfizer. The remaining authors declare no conflict of interests.
